# Perspectives of Black/African American and Hispanic Parents and Children Living in Under-Resourced Communities Regarding Factors That Influence Food Choices and Decisions: A Qualitative Investigation

**DOI:** 10.3390/children8030236

**Published:** 2021-03-18

**Authors:** Debbe Thompson, Chishinga Callender, Denisse Velazquez, Meheret Adera, Jayna M. Dave, Norma Olvera, Tzu-An Chen, Natalie Goldsworthy

**Affiliations:** 1 USDA/ARS Children’s Nutrition Research Center, Department of Pediatrics, Baylor College of Medicine, 1100 Bates Street, Houston, TX 77030, USA; Chishinga.Callender@bcm.edu (C.C.); himedenisse@gmail.com (D.V.); msa8@rice.edu (M.A.); jmdave@bcm.edu (J.M.D.); 2 Psychological, Health, and Learning Sciences Department, University of Houston, 3657 Cullen Boulevard Room 491, Houston, TX 77204, USA; nolvera@central.uh.edu (N.O.); tchen3@central.uh.edu (T.-A.C.); 3 HEALTH Research Institute, University of Houston, 4849 Calhoun Road, Houston, TX 77204, USA; 4 Common Threads, P.O. Box 163930, Austin, TX 78716, USA; ngoldsworthy@commonthreads.org

**Keywords:** minority, culinary, qualitative, photo-voice, parents, children, under-resourced, socio-ecological model, Black/African American, Hispanic

## Abstract

Families living in under-resourced communities are at risk of obesity and obesity-related chronic diseases. To develop effective interventions, it is important to identify parent and child perspectives of factors that influence food-related choices and decisions. This paper reports qualitative findings from a larger mixed method study investigating this topic. Hybrid thematic analysis was used to code and analyze the interviews. Family-generated photographs of factors influencing food choices were discussed during the interviews. Qualitative findings were organized by the socio-ecological model. Verbatim quotes and photographs were used to support themes. Thirty-six interviews were conducted (18 parents, 18 children). Findings from parents revealed personal (e.g., culture, beliefs, time), family (e.g., mother, child, father, health, finances, cohesiveness), environmental (e.g., availability, convenience, cost), and other (e.g., school food) factors influenced food choices. Similarly, child-reported influences were personal (e.g., preferences, beliefs, taste), family (e.g., mother, family encouragement, father, family time), social (e.g., school, friends), environmental (e.g., availability), and other (e.g., media, sports). The socio-ecological model provided a useful framework for identifying factors that influence food choices and decisions of families living in under-resourced communities. A deeper understanding of these factors could enhance both responsiveness and effectiveness of interventions to enhance diet and reduce obesity risk in families living in under-resourced communities.

## 1. Introduction

Prevalence of child obesity in the United States is at an all-time high, with an estimated 2017–2018 prevalence of 19.3% [1]. This is particularly true among children who self-identify as minority (25.8% Hispanic; 22.0% Non-Hispanic Black) [2] and those living in low-income households [3]. Similar trends have been observed in Texas (TX). In 2018–2019, 17.3% of TX youth ages 10–17 had obesity [4]. Compared to their White peers, Hispanic and African American children living in TX had nearly twice the rate of obesity [5]. Finding ways to effectively address the obesity epidemic and related disparities is a public health priority [6].

Evidence clearly establishes that multiple factors influence obesity risk [7,8]. Diet, through its role in excess energy intake, plays an important role in overall risk [8,9]. Similar to obesity risk, multiple factors influence dietary intake, including personal food preferences [10], the home food environment [11], food parenting practices [12], family meals [12], household income [13], and food marketing [14], among others. Understanding the ways in which these and other factors influence a family’s food choices is an important first step in the development of effective interventions.

Not everyone is influenced in the same way, however. For example, families living in low-income areas are more likely to be exposed to food marketing for less nutritious foods and beverages [15]. They are also more likely to live in communities that have a greater concentration of fast food restaurants and buffets [16] and less access to healthy food outlets with reasonably priced foods, such as fresh fruits and vegetables [17].

The Socio-Ecological Model (SEM) provides a framework for understanding the multiple levels that influence health behaviors [18] such as child obesity risk [19,20,21] or dietary behavior [22,23]. Ecological Models, such as SEM, view child obesity as a system, with multiple levels of influence [20,24,25]. The individual (intrapersonal level) is nested within and interacts with social (interpersonal) and community (environmental) domains. Understanding how these domains interact to influence food-related behaviors, and ultimately, consumption, is essential to understanding behavior and developing effective intervention approaches.

Within the SEM, family can also be viewed as a system operating within a larger system. Family Systems Theory posits that families are comprised of individuals who act independently, but who are also interconnected, where one member’s behavior affects others in the system [26]. Family is an important influence in a child’s life; family, particularly parents, is the source of many learned behaviors, such as those related to food, including preferences, beliefs, and dietary patterns [27]. Therefore, understanding multiple family members’ perceptions is important to gaining greater insight than could be obtained from interviewing only one family member. Dyadic research, i.e., conducting separate interviews with parent/child dyads, is one way to accomplish this [28].

Qualitative research offers a systematic method for understanding perspectives, meanings, and roles of individuals in real-world situations [29,30]. A strength of this type of research is that it acknowledges and conveys respect for the lived experiences of others [31]. It is also a flexible methodology that can be used in multiple ways, such as developing the item pool for a new survey instrument [32], designing intervention content [33,34], and understanding intervention effects [35]. Qualitative research has also been used to understand challenges faced by individuals in under-resourced communities, such as food pantry clients’ perceived barriers to making healthy food choices [36] and the perceptions about using farmers markets to purchase fruits and vegetables for their families among urban Special Supplemental Nutrition Program for Women, Infants, and Children (WIC) clients [37].

Photovoice is a qualitative approach that provides an opportunity for participants to “tell their story” in pictures [38,39]. It enables researchers to obtain a deeper, more nuanced understanding of an individual’s reality, challenges, and perspectives regarding a particular issue [38]. Photovoice has been used to understand perspectives on a variety of issues and topics, including the built and social environments [40], factors that influence body weight [39], dietary behaviors [41], and home food preparation practices [42].

Although it is well-documented that families living in under-resourced communities face numerous challenges obtaining and consuming healthy foods [13,43], little is known about the perceptions of parent–child dyads regarding factors that influence their food-related choices, decisions and behaviors from a SEM perspective. This information is critical for developing targeted, realistic, and appropriate interventions promoting healthy dietary choices. Thus, the purpose of this research was to address this important gap in understanding.

## 2. Materials and Methods

### 2.1. Aim

This research was part of a larger mixed method study with parent/child dyads living in under-resourced families in a large urban area in the Southwestern United States. Here, we present the qualitative findings reporting family perspectives of factors that influence their family’s food choices and behaviors. We also report parent-reported demographic and household characteristics to contextualize the findings.

### 2.2. Research Design

Qualitative methods (telephone interviews, Photovoice) were used to gather the data reported in this paper. Families were stratified by parent’s self-reported race/ethnicity (Black/African American, Hispanic) and family role (parent, child). Each member of the parent–child dyad participated in one interview. A total of 36 interviews were conducted (18 parent, 18 child). Prior to completing an interview, parents provided demographic and household information via an online survey hosted on a secure password protected website.

### 2.3. Setting

Families living in under-resourced communities in the Greater Houston, TX area were recruited for this study. To identify potential areas in which to focus recruitment efforts, zip codes of under-resourced communities were identified in the neighborhoods of Houston and surrounding areas. The City of Houston “Super Neighborhoods” website was a resource that indicated neighborhood demographic characteristics, including income, educational status, and race/ethnicity. The neighborhoods with a majority population of racial and ethnic minorities and/or lower socioeconomic status, by zip code, were included in a table. Recruitment efforts focused on families living in the zip codes included in the table.

### 2.4. Recruitment

Recruitment began in early May 2019, and ended in mid-August 2019. Recruitment methods included the volunteer database at the USDA/ARS Children’s Nutrition Research Center, Baylor College of Medicine, and word of mouth. Data collection began in June 2019 and ended in October 2019.

Eligibility criteria included: child 8–13 years old; parent/guardian; both parent and child fluent in English or Spanish; child receives and/or is eligible for free or reduced price lunch at school; healthy (i.e., no physical, health, or medical condition that would affect diet or study participation); parent and child living in same household; parent has primary responsibility for family food shopping/acquisition and/or meals; transportation to focus group (if participating in a focus group); families also needed to be willing to provide contact information, participate in study activities, and have focus group/telephone interview audio recorded. Further, families needed to reside in an area served by Common Threads (current or future), the organization that funded the project. We chose to focus the sample on 8–13 year olds because our past research indicates children this age can successfully participate in interviews [33,44,45]. Further, we chose to conduct separate interviews with parent/child dyads to obtain greater insight into factors that influence a family’s food choices and behaviors [28]. It is well-documented that although parents are the gatekeepers of the home food environment, children exert a strong influence [12,46] on foods available in the home environment. Therefore, a decision was made to conduct separate interviews with parent/child dyads to develop a more nuanced understanding of factors that influence a family’s food choices and decisions from both the parent and child perspective.

At the time of consent, parents were offered the opportunity for their family to participate in either focus groups or telephone interviews in their preferred language (English, Spanish). All parents chose for their family to participate in interviews; therefore, no focus groups were conducted. Language choice varied.

### 2.5. Procedures

Data sources included online surveys assessing demographic and household characteristics [36,47,48], family-generated photographs of situations that made it easy or hard to make healthy food choices, and telephone interviews.

Parents and children were given separate passwords to log on to the online survey to provide demographic and household information. The survey was hosted on a secure, password-protected server. Interviews were guided by a script developed by the research team; probes and prompts were used to expand, clarify, and understand responses. Separate scripts guided parent and child interviews. (See Supplementary Materials S1 [children] and S2 [parents]). Sample questions included: Parent—How can parents help their children to eat healthy?: Child—What or who influences you to try new foods? Families sent their photographs to the research team prior to the interview. Interviews were conducted by trained research coordinators. Interviews were digitally recorded, professionally translated to English (if conducted in Spanish), transcribed, and reviewed for accuracy prior to analysis.

At the end of the interview, the parent and child were asked to select a photograph that showed something that made it hard to make healthy choices; they were then asked to select a photograph that showed something that made it easy to make healthy choices. Following this, they were asked to select additional photographs they would like to discuss. Each photograph they selected was discussed using the “SHOWeD” mnemonic, a widely used Photovoice technique: 1—What do you see; 2—What is happening; 3—How does this relate to your food choices; 4—Why does this exist; 5—How can you be empowered by this; 6—What can we do about it? [39,49]. Probes were used as needed to expand, clarify, and understand their responses.

### 2.6. Analysis

#### 2.6.1. Surveys

English and Spanish language surveys were integrated into a single dataset. Separate datasets were maintained for parents and children. Descriptive statistics (frequencies, percentages) were calculated and reported for demographic and household data.

#### 2.6.2. Interviews

Final transcripts were analyzed by two trained and experienced independent coders using hybrid thematic analysis which uses both deductive and inductive methods to code transcripts [50]. A priori codes were based on the SEM and were used to initiate and provide structure to the coding process; emergent codes were generated during analysis to ensure the “voice” of the participants was represented. Analysis was conducted on English language transcripts (i.e., those conducted in English as well as those conducted in Spanish and then translated into English). Coders independently coded transcripts and then met routinely to compare coding and resolve differences. After all transcripts were coded, codes were reviewed and collapsed into themes based on the SEM. NVIVO 12 Plus (QSR International, Burlington, MA, USA) facilitated analysis. Findings from parent and child interviews were separately organized by levels of influence (i.e., SEM). A codebook was maintained throughout the analytic process. It included a priori and emergent codes; definitions; dates codes were added, removed, or collapsed into larger codes; and key decisions regarding coding decisions. As such, it served as a detailed audit trail of the analytic process.

#### 2.6.3. Quotes and Photographs

Verbatim quotes and photographs taken by the participating families were used to support findings. Direct quotes were italicized. To protect confidentiality while distinguishing quotes and demonstrating representativeness, the following coding system was used: P = Parent; C = Child; A = Black/African American; H = Hispanic. Note race/ethnicity designation in the coding system was based on how parent self-identified race/ethnicity at time of recruitment. A number (from 1–18) was randomly assigned to each parent–child dyad to help distinguish their quotes from those of other dyads.

## 3. Results

### 3.1. Recruitment

A total of 51 families were contacted; of these, 18 families agreed to participate in the study and provided written informed consent and child assent. The primary reasons for nonparticipation were disconnected phone numbers; no response to calls, texts, and/or emails after initial contact; scheduling conflict (e.g., vacation time); not meeting inclusionary criteria; and not returning consent packet.

### 3.2. Language

10 parents completed the survey and interview in English, and 8 completed the survey and interview in Spanish. 12 children completed the survey and interview in English; 2 completed the survey and interview in Spanish; and 4 completed the survey in Spanish and the interview in English.

### 3.3. Family/Household Characteristics 

All parents who participated in the study were female (100%) (hereinafter referred to as mothers), and most were 40–49 years old (61%), minority (56% Black/African American, 44% Hispanic), and married/living with significant other (61%). Mothers reported that slightly over half of the children participating in the study were female (56%) and Black/African American (56%). Fifty percent of mothers reported their child’s ethnicity was Hispanic. The majority received free/reduced price lunch at school (95%) (Table 1). Children ranged in age from 8–13 years of age, distributed as follows: 8–10 years old (22.2%); 11–13 years old (77.8%) (Data not presented in Table 1).

Household size varied, with one to five adults living in the home (including mother) and one to four children under the age of 18. The most frequently reported household size averaged four members, two adults (including mother) (39%) and two children under the age of 18 (50%). Household education varied; 33% of households had someone who had some college coursework, 22% had someone who held a college degree, and 11% had someone who held a postgraduate degree. Annual household income was $21,000–$41,000 (44%). Sixty-one percent of mothers reported their family spoke mostly English at home. Finally, most households had high/marginal food security (89%) and mothers reported relying on 1–3 food assistance programs (67%) (e.g., SNAP, WIC) to supplement their household food needs (Table 1).

### 3.4. Interview Findings

**Figure 1:**
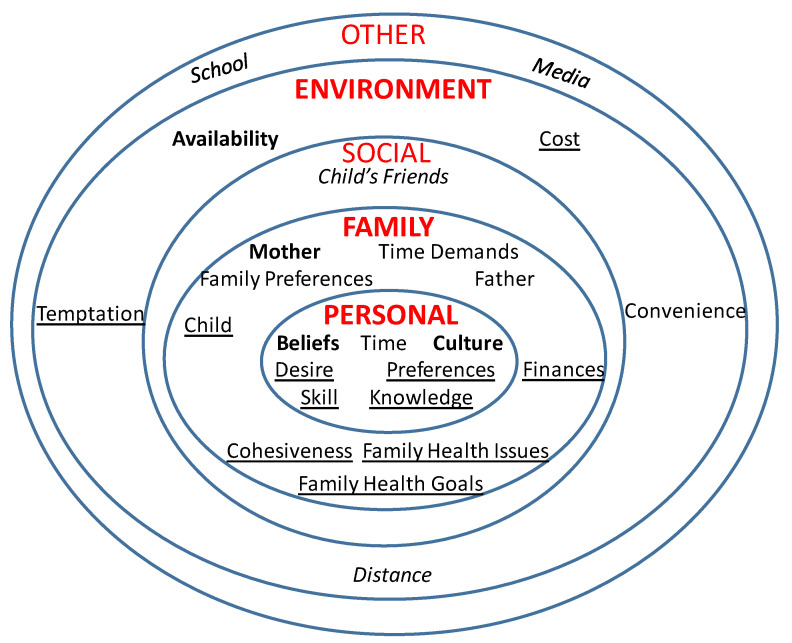
Mother’s thematic network. Legend: Majority of mothers mentioned– bold font; many/most mentioned—regular font; some mentioned—underlined font; few mentioned—italicized font.

**Figure 2:**
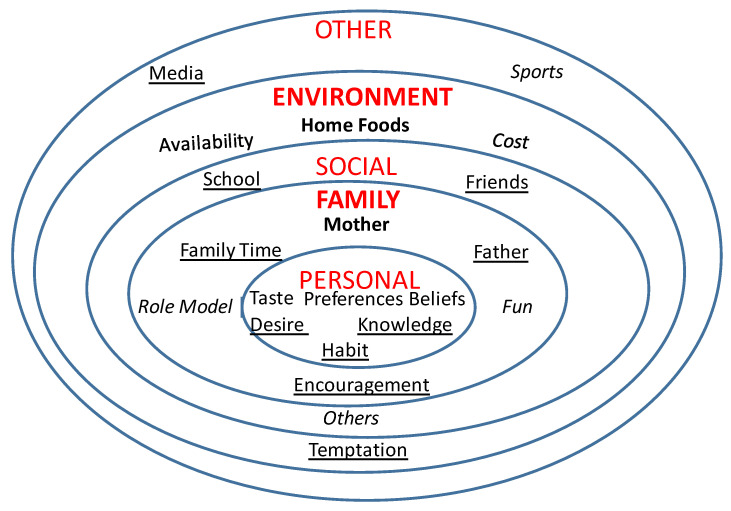
Children’s thematic network. Legend: Majority of children mentioned– bold font; many/most mentioned—regular font; some mentioned– underlined font; few mentioned—italicized font.

Child interviews averaged 43 min compared to parent interviews, which averaged 56 min. Using the SEM as a guide, parent and child responses were organized by personal, family, social, environmental, and other influences. Each influence and related themes are discussed in more detail below and supported by verbatim quotes and photographs taken by families. Within each influence, findings from parents are presented first, followed by findings from children. Themes are presented in descending order. The thematic networks are presented in Figure 1 (parents) and Figure 2 (children). A comparison of the parent and child themes within each level reported by the majority of participants is presented in Table 2. Theoretical saturation was attained separately for parents and children.

### 3.5. Personal Influences

**Mothers.** Personal factors that appeared to exert the greatest influence on mothers’ food-related behaviors were culture, beliefs, and time. Additional factors mentioned by some mothers were desire, personal preferences, skill, and knowledge.

**Figure 3:**
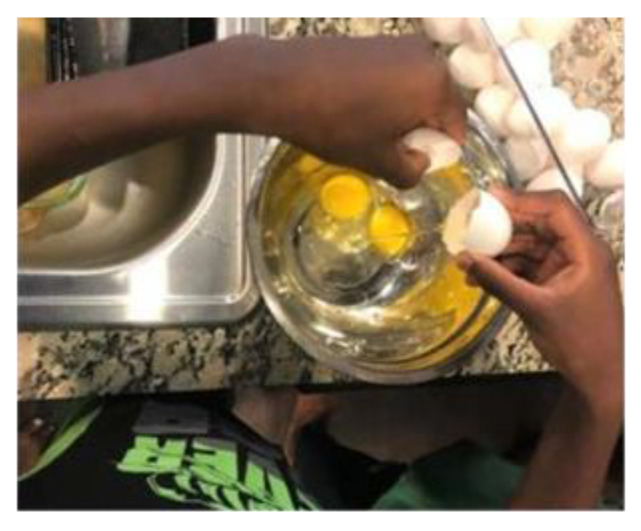
Child helping prepare breakfast (photograph submitted by family).

Culture. While culture was mentioned by nearly all mothers, it was viewed as having positive, negative, and variable effects on food-related behaviors. Some mothers said the way they were raised had a positive influence on the way they parent around food: *I think a good breakfast to start the day would be good….my mother did it for me, I don’t know, it is something that I was brought up with….I feel like my kids have a good start. I’ve done the bare minimum to start them off correct* (P.5A). (Figure 3).

Other mothers said their culture had a negative influence on food behaviors: *My culture…is not that healthy. We eat a lot of junk food…crackling pork skins with too much fat, very greasy food, children don’t like fruit, vegetables. It’s very difficult to their food habits* (P.6H).

Others stated culture had a variable effect: *I’m African American, so I grew up eating a lot of fried food and high fat foods. We would gather to eat for Sunday dinner, things like greens, cornbread, macaroni and cheese… culture plays a role, and if you’re taught to eat that way and then you’ll be more likely to continue that behavior. But if your culture is something where health is considered and certain things are not even in the home, then you’ll have a healthier way of looking at food, even where I grew up. Yes, culture does influence the way you eat, I believe, whether it’s negative or positive* (P.16A).

Beliefs. Almost all mothers expressed beliefs that influenced food-related choices and decisions. Some believed that certain foods, such as canned and frozen foods, flour, pasta, pork, and red meat were unhealthy. As one mother said: *So, if the dish calls for animal protein, I almost never give my kids animal protein. In reality, that would be a problem for me, because I don’t think it’s necessary to eat red meat all the time or even often. You can replace it with vegetables that also contain a high amount of plant protein* (P.17H).

Although eating healthy foods was believed to be important, it was also believed to be expensive: *It seems the healthier you eat the more the food costs: If it was available, I think if people had access to healthy foods that cost just as much as the regular foods they are buying, they would be more open to purchasing them* (P.16A).

Some mothers also expressed misconceptions about what constituted a healthy food. For example: *…the fries at Chick-Fil-A has some small holes and squares, and I tell them [children]—“Those holes are so that the oil is removed from the fries”, so you’re not eating that much grease* (P.10H).

**Figure 4:**
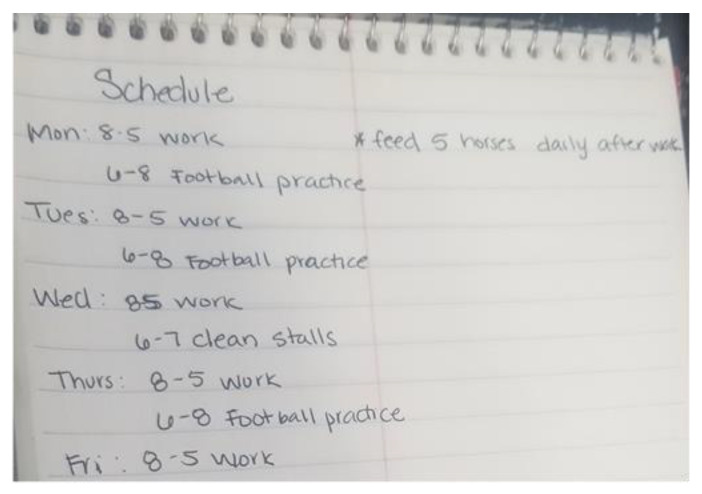
Family’s weekly schedule (photograph submitted by family).

Time. Similar to culture, time was seen as having negative, positive, and variable effects by many mothers. Limited time or demanding schedules were most often mentioned. For example, one mother provided a photograph of the family’s weekly schedule. (Figure 4) Between her work schedule and the child’s extra-curricular activities, it was difficult to eat a healthy meal at home: *I did a copy of our schedule, our weekly schedule at home. Because a lot of times…well I’m not going to say a lot of times, but sometimes it’s hard to prepare a healthy meal or even eat a healthy meal because our schedule is so tedious. And you know it’s a lot of time consuming, a lot of driving. And so with my son playing sports, he has practice three days a week. So sometimes we don’t have the time to actually sit down and have a healthy meal. You know we’ve got to get it on the go* (P.9A).

A few mothers who were not currently working outside the home did not believe time was a problem: Time? No, not right now because the children are on vacation. I’m here with them, I don’t work. So, of course I can go to the store to buy the ingredients or whatever I need. No, time is no obstacle (P.14H). Others believed time had variable effects on food consumption: *Time? Sometimes, not all the time. So, for example, I’m always running around with the kids, school, house chores, so that could vary, you understand? It could be time; it could be something else. I don’t know* (P.7H).

Other influences mentioned by some mothers were personal preferences (*When I’m trying to watch what I’m eating, like when I’m—I like cranberries. I like nuts. I mix them in my own trail mix*—P.7A), desire (i.e., motivation) (*I want to be healthy, and I want healthy children*—P. 16A), *skill* (*I’m an ok cook but not like a great cook*—P.11A), and knowledge (*I didn’t know the grains meant rice*—P.8A).

**Figure 5:**
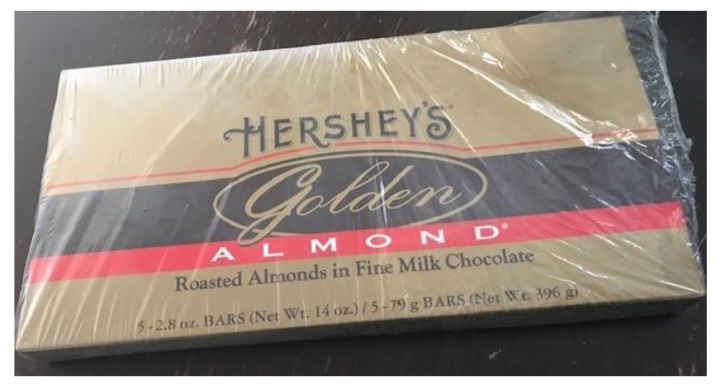
Child’s favorite snack (photograph submitted by family).

**Children**. Personal influences on food behaviors mentioned by many children were preference, beliefs, and taste. For example, one child, who loved chocolate, believed choosing a chocolate bar with nuts made it a healthy snack. (Figure 5) When asked how it felt to make this choice, the child responded: *It makes me feel I guess okay… or more okay with my choices than just being bad* (C.2A). Some children also reported their eating behavior was influenced by desire (i.e., motivation), habit (…*even though I like more healthy eating like I like fruits and vegetables and stuff it’s just I always find myself eating more unhealthy foods*—C.15A), and knowledge.

### 3.6. Family Influences

**Mothers**. The strongest influence on family food behaviors reported by nearly all mothers was themselves (mothers). Many also mentioned time demands, family preferences, and fathers. Some also mentioned children, family health goals, finances, cohesiveness, and health issues.

Mothers as strongest influence on family food behaviors. Mothers viewed themselves as having a substantial influence on their children’s food behaviors: *…it is important for me to give healthy food to my children. I try not to let them eat street food, to eat always at home, so that they can always be healthy* (P.3H). They also viewed themselves as a role model for their children: *Lead by example, I always tell my kids, I like to lead by example. I may be the only example you see that’s doing what’s right and what’s wrong* (P.8A).

Time demands. Time demands was mentioned by some mothers as an influence on their family’s food behaviors. Work and school schedules and outside family activities seemed to be particularly influential. As one mother said: *My job does not allow me the time to cook* (P.16A). Another said: …*one gets busy, you’re in a hurry, on the run, the easiest thing to get is fast food, and you tell yourself: “I’ll just go and grab something. We have to eat”* (P.7H).

Family food preferences. Food preferences, particularly those of children, appeared to influence the home food environment from some mothers’ perspective. While some children’s preferences represented a challenge *(My son child doesn’t eat vegetables*—P.6H), other children made it easier for their mothers to serve healthy foods: *She’s one of those kids that wants to try stuff* (P.8A). Despite the challenges, however, some mothers were determined to provide healthy foods to their family: *I try to include vegetable dishes, even if sometimes they don’t eat them. I struggle with that, especially with the children. But regardless, I do try to make it healthy and the food fresh* (P.12H).

Fathers’ influence. Some mothers reported fathers influenced the family’s food-related activities. However, fathers were viewed as having a variable role. Several viewed fathers as having a negative influence on the home food environment: *He comes home from work and hopes to arrive and see a warm, fatty meal... because he craves that more. I struggle a lot with him because he wants very greasy food. I struggle a lot with him* (P.14H). While several mothers said fathers helped with food preparation, others did not. One mother described her husband’s role as “occasional helper or occasional assistant” (P.13A), and another mother mentioned her husband “cooks sometimes” (P.5A). Food-related activities appeared to be mostly the responsibility of the mother.

Children’s influence. Some mothers said children influenced family food behaviors. A few mentioned their child liked to cook (*…and sometimes a lot of times my son cooks…and my …daughter, too* (P.4A). Others mentioned ingrained food patterns were difficult to change (…*they get bratty because: “I don’t want that, I’m not eating”, just like that. So, yes, I say there are many things. And let me tell you, history repeats itself because they’re growing up just like one did, giving them food that you think it’s healthy, but in the end you realize that it’s not that healthy, but they’re used to eating that way. And it’s difficult to change that behavior* (P.7H).

Family health goals. Some mothers reported that family health goals influenced food-related behaviors. There seemed to be a particular interest in preventing negative health outcomes: *Of course so that all of us can be healthy. So you know and to avoid that you know to avoid things like high blood pressure that my husband had some time back, so that it can come down. Yeah that’s why I’m keen on avoiding all those sodas and the sugars and too much salt and junk and all that. It’s very important to me* (P.2A). *I try to buy a lot of lean meats, so my goal is to continue providing well-balanced meals for my children. I guess to avoid obesity and the health problems that come along with it* (P.16A).

Finances. For some, family finances also influenced foods consumed in the home. As one mother said: *Sometimes... you don’t eat healthy at home... because our budget is really low; but, we try to do the best we can* (P.3H). Another mother said finances influenced purchases made at the grocery store: *Whatever I’m purchasing is based on what they offer….they don’t offer everything of course* (P.13A).

Family cohesiveness. Another concept that described food-related activities in some families was family cohesiveness (i.e., togetherness). For example, some saw food-related activities as a family event: *Whenever we do something, we always agree on who is going to take what. We all take something and we all take different things* (P.3H). For others, changes to the family diet were made together: *We changed the mayonnaise because we figured out that the vegan was healthier, so we changed it. We all decided to try it and it sure is healthier* (P.10H). Others saw food preparation as a time for demonstrating love and for family bonding: *I mean it makes my family happy. I feel it makes them feel loved when I cook. I work all day and I come home and prepare them food, and it actually gives us time to bond. They are all downstairs, looking at me as I cook and we are interacting* (P.16A).

Finally, some mothers reported that family health issues, such as a father’s diabetes or child’s food allergies, impacted food consumption and home availability.

**Children**. Similar to findings that emerged from interviews with mothers, nearly all children reported their mother influenced their food-related behaviors. As one child said: *I like to walk to the store and buy chips, but my mom told me that I shouldn’t be walking to the store and getting chips because chips are unhealthy, so she just doesn’t give me the money. She says if you go to the store you better come back with some fruit cups or something* (C.1A). Some children reported that family encouragement, their father, and family time influenced what they eat: *In my household, my family, like my sisters, especially my older sister, my dad, my mom, they really like encourage us to eat foods that don’t contain things that are bad for your body and healthy things* (C.10H). A few also reported that their family served as role models and make food-related activities, such as cooking, fun: *When we’re in the kitchen together and I make mistakes they help me and we try to fix it and that we have fun while we’re in the kitchen too* (C.16A).

### 3.7. Social Influences

**Mothers**. Few mothers reported social influences external to the nuclear family exerted a strong effect on their family’s food intake. As one said, *there is too much junk in the market and you know it’s just easily available and you know so it’s hard to feed your family healthy or to get—because their friends are eating all these things* (P.2A).

**Children**. Similar to mothers, children reported few social influences on their eating behaviors. School and friends were mentioned by some as having both positive and negative effects. For example, one child said: *School is a good example…they give us balanced meals there* (C.14H), while another said: *There’s not as much good food [in school] like good vegetables* (C.2A). Friends were also seen as having positive and negative influences on their eating behaviors. A few children also mentioned other people in general as having an influence on their food-related behaviors: *You see like somebody buy a lot of something you might want to try it because you know, since they’re buying a lot, it must be good* (C.15A).

### 3.8. Environmental Influences

**Mothers**. Availability, convenience, cost, temptation, and distance were mentioned as environmental influences on family’s food behaviors.

Availability. Nearly all mothers reported availability influenced their family’s food behaviors. For example, some talked about the importance of having healthy foods available in the home (e.g., fruit washed and ready to eat; healthy meals): …*and with fruit, I try to keep as many containers full of fruit as possible, so that they don’t have to wash them, so that they can see it appetizing there, in the refrigerator* (P.6A). Others mentioned the effect of not having healthy foods readily available: …*And so when they get home, the parent hasn’t cooked so they have to rely on the junk food or whatever the parent has supplied them* (P.4A).

**Figure 6:**
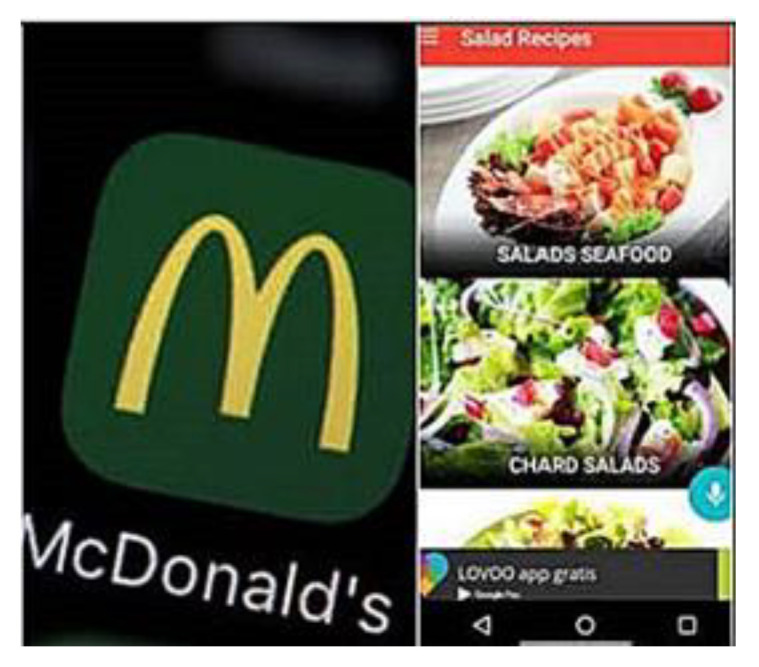
Mobile food apps (photograph submitted by family).

Convenience and cost. Some mothers mentioned convenience of living close to fast food establishments and cost: *We already have easy access to fast food, what do we do? We opt for already cooked food, for the easiest. And as in some places they give us accessible prices, which turns out cheaper... to better grab things from the one-dollar menu than cook and one does it because of the lack of time and the fact that the food is already done, one does not want to cook either, it makes things easier* (P.12H). Fast food apps were also mentioned:* McDonald’s app, I think that that makes it hard to eat healthy because the apps are on our phones. It’s just a push of a button: you click on what you want and someone will bring it to you or you can drive right up to the McDonald’s and they bring it out to your car* (P.15A). Interestingly, this mother also provided a photo of an app for Salata (i.e., a fast-casual restaurant chain featuring made-to-order salads and wraps) representing a healthier option that was also convenient. (Figure 6).

**Figure 7:**
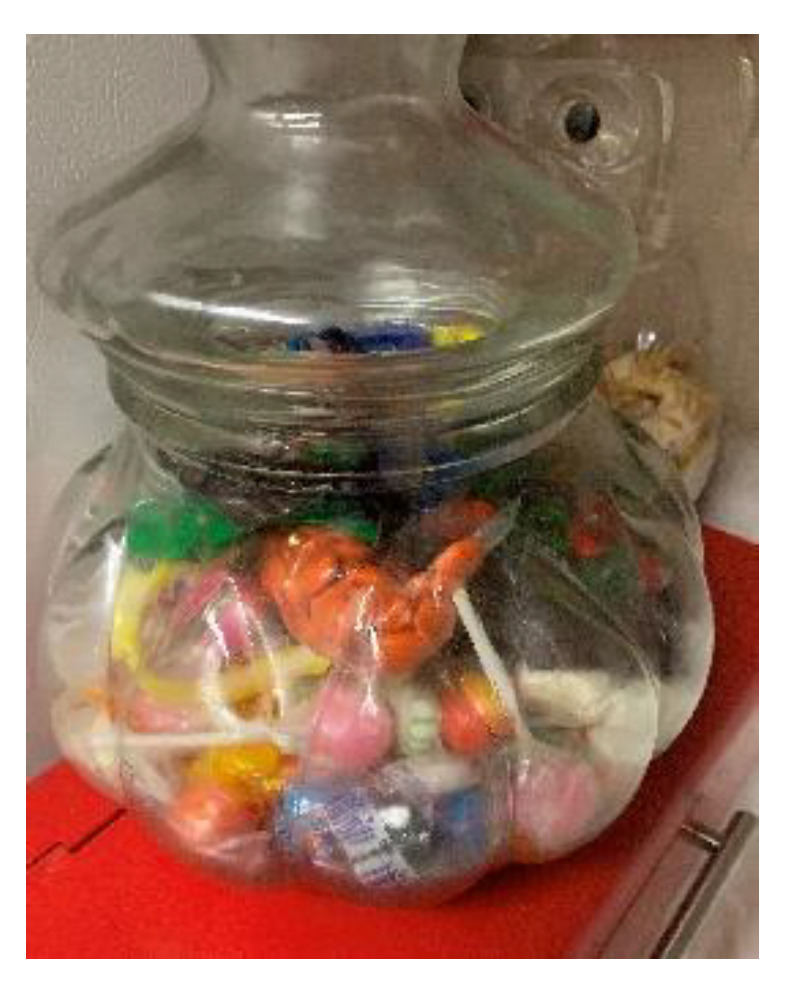
Candy jar on kitchen counter (photograph submitted by family).

Temptation. Some mothers also mentioned the effect of temptation on eating behavior. For example, one mother talked about the family tradition of having a jar of candy on the counter as a source of temptation that makes it hard to eat healthy: *The candy jar….It’s sitting there waiting for someone to get a candy out of it. It’s a bad choice; it’s readily available versus maybe instead of having the candy jar out maybe having a fruit bowl there. It’s kind of like family tradition with me. My family always had candy jars.* (P.15A). (Figure 7) A few also mentioned that distance from healthier options influenced foods eaten at home.

**Children.** Nearly all children said foods in the home influenced their food behaviors, while many others discussed availability more generally. As one child said: *When you have the things that you need to eat at home you can easily grab them or you could easily go to the store and go grab some healthy food that you need* (C.8A). Some children said temptation influenced their food choices, while a few also mentioned cost.

### 3.9. Other Influences

**Mothers**. A couple of mothers mentioned media influenced their family’s eating behaviors. While one thought media was a positive influence, another thought media advertisements had a negative effect on food behaviors. A couple of mothers also mentioned foods consumed at school. One felt she had no control over what her children ate at school, and one did not agree with the foods served in schools.

**Children.** More children than mothers believed the media influenced food behaviors. Some children also said the media influenced their food behaviors in both positive and negative ways. A few children also mentioned sports (e.g., football, gymnastics) influenced their eating behaviors. For example, one child stated: *When I got to third grade, I started eating a lot because I started playing football more, so I wanted to get bigger* (C.1A).

## 4. Discussion

This research demonstrated that the SEM provides a useful framework for understanding the multiple levels of influence on dietary behaviors and choices from the perspectives of parents and children living in under-resourced communities. These insights may make important contributions regarding the design of dietary change interventions for families living in under-resourced communities.

The recognition that behavior is influenced by multiple factors both within and external to an individual is well-documented in the literature [51,52,53]. Models such as the SEM [19,20,21] also provide a useful framework for investigating the factors that influence complex behaviors such as diet [12,54] and child obesity risk [19,20,21]. Identifying these factors in under-resourced populations and understanding how they influence behavior is an important step in the design of effective interventions [55]. However, a recent literature review found that only 23% of the studies investigated obesity-related behaviors in children on three or more levels of the SEM [56], despite consensus that it is an appropriate model for understanding child obesity [21]. This may partially explain why interventions often have mixed effects when attempting to change diet [21,57].

In qualitative research, the ultimate goal is to “tell the story” of those who participated in the research in relation to the specific topic or question of interest [58]. True to that goal, in this study it was clear that both mothers and children wanted to make healthy food choices, but certain factors hindered their ability to do so. In particular, although multiple factors were identified at all levels, various factors at the family and environmental levels were mentioned by the majority of parents and children. This finding is consistent with current scientific understanding regarding the complexity of factors influencing a behavior, particularly those associated with obesity [9,51]. It is also consistent with Family Systems Theory which posits that there is a reciprocal interaction between the individual and the family [26]. Future research could further investigate these levels using an approach informed by Family Systems Theory to more thoroughly understand the subtle ways in which the personal and family levels interact to influence a family’s food choices.

For mothers in this sample, beliefs and culture were the strongest personal influences on food related behaviors. Similar to our sample, beliefs that canned or frozen foods are less healthy [59] and that eating healthy is expensive [36] have been reported by other families with limited income. These findings suggest that interventions targeting low-income families should emphasize the healthiness of canned and frozen foods as well as purchasing (e.g., frozen fruit or vegetables without added ingredients such as sugar or sauces) and preparation techniques (e.g., rinsing canned vegetables or pulses prior to use) to reduce undesirable additives such as sugar, fat, and salt.

Interestingly, culture was found to have mixed effects on food choices. While some mothers reported culture had no influence, others reported that it had negative or variable effects. These findings were inconsistent with the findings of others who reported that culture has a strong effect on food-related behaviors [60]. Previous research has suggested the influence of culture is particularly salient for minority families [61,62,63,64,65]. Alternatively, mothers of Black/African American children reported that while culture did not influence their everyday food behaviors, it did influence their diet and food choices on specific occasions (e.g., Sunday dinner, holidays); some mothers also reported they found healthier ways to serve traditional foods [66]. Other researchers have reported that acculturation appears to influence dietary behaviors of minority families [67]. This suggests that additional research is needed to more fully explore this topic and to understand potential effects of culture, identity, and acculturation on food related choices and behaviors, as well as culturally sensitive and relevant intervention strategies.

Unlike mothers, children reported that taste and preference influenced their food related behaviors. This is not surprising, given the large body of evidence supporting that children’s food choices are influenced by both taste and preference [10,68]. While certain aspects of taste may be genetically programmed or influenced by in utero exposure to mother’s diet, preferences are thought to be learned through a complex interplay of genetics, physiology, environment, and exposure [68,69,70,71]. While it is not likely that innate food preferences can be modified, evidence suggests that preferences can be influenced by the gradual introduction of new foods on multiple occasions [72] in developmentally appropriate ways [73]. This suggests that interventions to modify the food related behaviors of children should include strategies for parents on ways in which to improve preferences, including what to expect from children, modeling, and the importance of persistence. Future research could be conducted to more fully explore this topic from the perspectives of both parents and children, with the goal of identifying potentially effective intervention strategies and approaches.

At the family level, both mothers and children indicated that mothers were an important influence on their child’s food choices, while fathers were viewed as playing a lesser role. The literature supports that mothers exert a strong effect on the eating behaviors of their children; however, less is known about father’s effect on child diet or food-related behaviors [12]. Work by Vollmer et al. [74] revealed that neither child diet quality nor body weight were associated with paternal feeding behavior (practices, style). However, paternal feeding behavior appeared to moderate the relationship between a child’s food-related behaviors and weight status. Therefore, our findings support that additional work is needed to more fully understand the father’s role on the home food environment and child eating behaviors. Alternatively, mothers revealed that children also exerted a strong influence on foods available in the home. This is consistent with findings reported by others [75]. This finding is also consistent with research indicating the family is a system where each member influences the behavior of others [12].

The environment was thought to be a strong influence on food choices and behaviors by both mothers and children. Availability appeared to be particularly strong influence from the perspective of both. Story et al. [76] published a conceptual model with which to view the ways in which various types of environment (e.g., home, school, community) influence food behaviors, particularly in minority populations. Given the multiple environments in which families live, work, and play, interventions designed to influence food-related behaviors of families living in under-resourced communities should include strategies for making healthful food choices in various environments.

In conclusion, this work adds support to the body of research calling for family-based child obesity prevention interventions [77,78]. Findings also add support for the importance of taking the broader context into consideration when designing child obesity prevention strategies and programs for families [19,25] and for adapting interventions for a specific population [25]. Finally, it speaks to the importance of working in partnership with families to understand an issue from their perspective [30]. In doing so, we move closer to leveling the playing field and attaining health equity [79].

Limitations of this research include a small sample size and conducting the research in a limited geographic region of the United States (a large urban area in the Southwestern US). A volunteer sample was recruited, meaning that the dyads who participated in this study may not be representative of families living in under-resourced communities in our area. Data were also not coded for racial or ethnic differences (Black/African American, Hispanic), and no fathers participated. These limitations must be examined with consideration that the goal of qualitative research is theoretical saturation, rather than sample size. Further, the study has several strengths that should be emphasized: use of two trained coders acting independently, an a priori research question which guided both data collection and analysis, inclusion of parent/child dyads, and focus on both Black/African American and Hispanic families living in under-resourced communities. Further, although the sample size (*n* = 36) was small for a quantitative study, it is considered adequate/sufficient for a qualitative study, ref. [80] supporting that theoretical saturation was attained and that reactions were independent of race or ethnicity.

## 5. Conclusions

This research demonstrated that the SEM provides a useful framework for understanding the multiple levels of influence on dietary behaviors and choices from the perspectives of parents and children living in under-resourced communities. In particular, it supports that interventions need to address multiple levels of influence in order to be effective at changing behavior. Consistent with the SEM, it also emphasizes the dynamic interplay between parents and children and how each influences the foods available in the home. These insights are important contributions to the design of dietary change interventions for families living in under-resourced communities and emphasizes the need for multi-level family-based interventions that work with the family unit, particularly the mother/child dyad, to modify food behaviors.

## Figures and Tables

**Table 1:** Family/household characteristics.

		*n*	%
**Parent (*n* = 18)**			
Age (years)			
	30–39	4	22.2
	40–49	11	61.1
	50–59	2	11.1
	≥60	1	5.6
Gender			
	Male	0	0.0
	Female	18	100.0
Hispanic			
	Yes	8	44.4
	No	10	55.6
Race			
	Black/African American	10	55.6
	White	6	33.3
	Other	2	11.1
Marital Status			
	Married/living with significant other	11	61.1
	Single, never married	3	16.7
	Divorced, separated, widowed	3	16.7
	Other	1	5.6
**Child (*n* = 18)**			
Gender			
	Male	8	44.4
	Female	10	55.6
Hispanic			
	Yes	9	50.0
	No	9	50.0
Race			
	Black/African American	10	55.6
	White	6	33.3
	Other	2	11.1
School lunch			
	Receives free school lunch	12	66.7
	Receives reduced price lunch	5	27.8
	Pays full price for school lunch	1	5.6
**Household**			
Number of children < 18 years old in home			
	1	1	5.6
	2	9	50.0
	3	7	38.9
	4	1	5.6
Number of adults in home, excluding you			
	0	2	11.1
	1	7	38.9
	2	5	27.8
	3	3	16.7
	4	1	5.6
Highest household education			
	Some high school	2	11.1
	High school graduate/GED	1	5.6
	Technical school	3	16.7
	Some college	6	33.3
	College graduate	4	22.2
	Post graduate study	2	11.1
Average annual household income			
	<$21,000	4	22.2
	$21,000–$41,000	8	44.4
	$42,000–$61,000	5	27.78
	>$61,000	1	5.6
Food security			
	High/marginal food security	16	88.9
	Low food security	1	5.6
	Very low food security	1	5.6
Parent food assistance program * usage			
	0 program participation	6	33.3
	1–3 programs	12	66.7

* Supplemental Nutrition Assistance Program (SNAP/food stamps); Women, Infants and Children (WIC); Farmers’ Market Nutrition Program (FMNP); Senior Farmers’ Market Nutrition Program; Summer Food Service Program; Child and Adult Care Food Program (CACFP); Food Assistance for Disaster Relief; Schools/Child Nutrition Commodity Programs; Food Distribution on Indian Reservations; Commodity Supplemental Food Program; The Emergency Food Assistance Program; Other.

**Table 2:** Themes mentioned by the majority of parents and children within each level of influence.

Level	Parent	Child
Personal	Beliefs	
	Culture	
Family	Mother	Mother
Social		
Environment	Availability	Home foods
Other		

## Data Availability

The datasets generated and/or analyzed during the current study are not publicly available due to concerns regarding privacy but select data are available from the corresponding author on reasonable request.

## Supplementary Materials

The following are available online at https://www.mdpi.com/2227-9067/8/3/236/s1, File S1: Child interview script. Semi-structured interview script showing questions and prompts that guided child interviews; File S2: Parent interview script. Semi-structured interview script showing questions and prompts that guided parent interviews.
