# Supplementary material for: Perspectives of Black/African American and Hispanic Parents and Children Living in Under-Resourced Communities Regarding Factors That Influence Food Choices and Decisions: A Qualitative Investigation
Source: Children (Basel). 2021 Mar 18;8(3):236. doi: 10.3390/children8030236 (PMC8002848; doi:10.3390/children8030236)
Supplement: Supplementary file 1 [file children-08-00236-s001.pdf]

S1: Child Interview Script

Introduction

Thank you for talking with us today. My name is [insert interviewer's name]. I am from the Children's Nutrition Research Center at Baylor College of Medicine.

Our research team was funded by Common Threads, an organization that provides cooking and nutrition education classes to families. The information you share with us will help them make sure their classes are useful and appealing to families. Therefore, we want to learn more about your thoughts. To do this, I will be asking you questions. There are no right or wrong answers to any of the questions we will ask. It's important that we hear what you think because everyone's opinion is important and your experiences may be different from what others think. We will audio record our session so we don't forget what is said today. Therefore, we ask that you speak clearly and avoid saying your real name out loud or refer to others' by their real name. This will help keep the information you share private. We will not use your real name in papers or presentations that we write on what you and others share with us.

1. Let's start by talking about eating healthy. What does "healthy eating" mean to you?
   Prompts:
   a. Example of healthy eating. *Probe for examples (food/beverages, people, places – i.e., home, school, etc.).*
   b. Example of unhealthy eating. *Probe for examples (foods/beverages, people, places – i.e., home, school, etc.).*
   c. What are reasons someone would eat healthy? Unhealthy? *For each, probe for rationale/justification, motivators e.g., taste, convenience, health, etc.*
   d. What happens to your body when you eat healthy foods? Unhealthy food? *For each, probe to identify benefits and consequences.*
   e. What or who influences you to try new foods? *Probe to identify role of family, friends, media, school/teachers, etc.*

2. On a scale of 1 (hard), 2 (not hard or easy), 3 (easy), how easy or hard is it to eat healthy?
   a. Ask for reasons for each rating. *Probe to identify obstacles and resources (i.e., barriers, facilitators) related to home, community, school, cultural practices/values, access, cost, etc.*
   b. What grade would you give your eating habits (i.e., A, B, C, D, F like in school grades)? Ask reasons for grade they gave. *Probe to identify ways to improve grade (for B or below).*

3. How could your parents help you make healthy food choices at home?
   a. What types of food do you usually eat at home (i.e., American, Hispanic, fast food, frozen foods, packaged dinners, vegetarian, etc.). *Probe to identify specific examples of foods and cuisines eaten at home.*

4. What does the word "cooking" mean to you?

5. In your opinion, is there a relationship between healthy eating and cooking skills? *Probe to understand their response.*

6. On a scale of 1 (not confident), 2, (not sure), 3 (confident), how confident are you in your cooking skills? *Probe to explore their reasons for the rating they gave.*
    a. On a scale of 1 (not important), 2, (not sure), 3 (important), how important is it to learn to cook? *Probe to identify reasons for their ratings.*
    b. What are examples of healthy cooking? *Probe to identify foods, cooking methods.*
    c. What are some examples of unhealthy cooking? *Probe to identify foods, cooking method.*
    d. How many of you have taken a cooking class with your parents? *Probe what it was like, what could have made it better.*

7. Do you cook meals with your parents? On a scale of 1-3 (1=do not like; 2=do not like or dislike; 3=like), how much do you like to cook meals with your parents? *Probe to identify reason for rating they chose.*
    Prompts:
    a. Their favorite foods or meals to cook with parents.
    b. Their role when they cook with parents (i.e., what do they do/help with).
    c. Role technology plays when cooking with parents; role technology COULD play when cooking with parents.

8. Do you go grocery shopping with your parents?
    a. [If yes, ask]: When you go grocery shopping with your parents, what is your role? *Probe to identify if they help choose foods like favorite fruit or vegetable, help choose foods for meals/snacks, etc.*

9. [A graphic of MyPlate will be emailed to their family before the interview.] Please take a look at "attachment 1" on the email I sent your family before our interview today. Can tell me what this is?
    a. What is it for? *Probe to identify understanding – i.e., can they identify the different parts and how to use them?*
    b. How many of you use it? *Probe how to use it; benefits and barriers to using it.*

10. [A graphic of a food label will be emailed to their family before the interview.] Please take a look at "attachment 2" on the email I sent your family before our interview today. Can you tell me what this is?
    Do you:
    a. Read food labels? *Probe to identify whether they can identify the parts of a food label.*
    b. Use food labels? *Probe how use it; benefits and barriers to using.*

11. Now, we are going to do something a little different. Take a look at the pictures you and your child took for this project. In the next half of our discussion, we are going to talk about your pictures. Take a look at the pictures. For each picture you choose, tell me the number and title you gave it. Now:
    a. Choose one that shows something that makes it hard to eat healthy.
    b. Choose one that shows something that makes it easy to eat healthy.
    c. (if time) Are there other pictures you would like to show us?

For each picture, ask the following six questions:

- 1-What do you see?
- 2-What is happening?
- 3-How does this relate to your food choices or health
- 4-Why does this exist (happen)?
- 5-How can you be empowered by this (how does it make you feel)?
- 6-What can we do about it?

12. Before we end, I have one more question for you. If you could design a program for someone your age to help them eat healthy and learn to cook:
    a. What would you include in it?
    b. What would you not include?
    c. What types of foods/recipes would you include? *Probe to identify specific foods, cuisines.*
    d. Who would it be for – i.e., who should come? *Probe parents, children, ages, etc.?*
    e. How long would it be?
    f. How would you let families know about it?
    g. What should happen at the last session (i.e., cook off with judges and a winner; celebration; certificates; etc.)?

CLOSING STATEMENT

Thank you so much for speaking with me today! Before we finish, is there anything else you would like to add? [Note: If yes, discuss with them; use probes and prompts as needed to clarify, understand, and expand their responses].

I will mail your family a $[insert amount] [state type of compensation-e.g., money order, cash] as a thank you for your time.

[After the interview is over, and you hang up the phone, please record your impressions of the interview, including anything that may have influenced the quality of the data (e.g., interruptions, crying children, bad connection, disinterest, etc). Please write the information below and then audio record it at the end of the interview recording; be sure to identify them as "interviewer debriefing" before you start talking.]____

S2: Parent Interview Script

Introduction:

Thank you for talking with us today. My name is [insert interviewer's name]. I am from the Children's Nutrition Research Center at Baylor College of Medicine.

Our research team was funded by Common Threads, an organization that provides cooking and nutrition education classes to families. The information you share with us will help them make sure their classes are useful and appealing to families. Therefore, we want to learn more about your thoughts. To do this, I will be asking you questions. There are no right or wrong answers to any of the questions we will ask. It's important that we hear what you think because everyone's opinion is important and your experiences may be different from what others think. We will audio record our session so we don't forget what is said today. Therefore, we ask that you speak clearly and avoid saying your real name out loud or refer to others' by their real name. This will help keep the information you share private. We will not use your real name in papers or presentations that we write on what you and others share with us.

Are there any questions before we get started?

Meaning of healthy eating and relationship to cooking

1. Let's begin by talking about healthy eating. What does "healthy eating" mean to you?
   Prompts:
   a. What's an example of healthy eating?
   b. What's an example of unhealthy eating?
   c. On a scale of 1-3 (1=not important, 2=not sure, 3=important), how important is eating healthy to your family. *Probe to understand reason for the rating they chose; explore family values/goals related to healthy eating.*
   d. Role of culture in healthy/unhealthy eating. *Probe to identify specific examples of the ways in which culture influences food behaviors.*
   e. Role of environment/community in healthy/unhealthy eating. *Probe to identify specific examples of influences.*
   f. What would you like to learn about healthy eating? *Probe to identify specific topics.*
   g. How would you like to learn this (i.e., nutrition education methods). *Probe to identify ideal nutrition education methods – in-person, group, online, written information, etc.*
   h. How many of you have attended a class or program on healthy eating? *Probe to identify where, approximately when, if a child attended with them, what they learned, and if they still use what they learned. If they have attended a Common Threads program, ask what they thought about it.*

2. How can parents help their children eat healthy?
   Prompts:
   a. Facilitators (e.g., modeling, availability, etc.).
   b. Barriers (e.g., time, cost, convenience, family preferences, child/family activities, availability, accessibility, etc.).
   c. Comfort level in helping their children eat healthy; what would increase their comfort level.

3. In your opinion, is there a relationship between healthy eating and cooking skills?
   Prompts:
   a. How many of you prepare (i.e., cook) meals for your family? *Probe to identify reasons for cooking/not cooking; types of foods prepared (e.g., American, Hispanic/Latino,* frozen foods, packaged dinners, vegetarian*); spouse/partner's role.*
   b. On a scale of 1 (not confident), 2, (not sure), 3 (confident), how confident are you in your cooking skills? *Probe to explore reasons for rating and what would increase their comfort level.*
   c. Using the same scale, how confident are you that you can cook healthy meals? *Probe to explore their reasons for rating and what would increase their confidence.*
   d. What are examples of healthy cooking? *Probe to identify foods, cooking methods.*
   e. What are some examples of unhealthy cooking? *Probe to identify foods, cooking methods.*

4. Do you let your child help you cook meals at home?
   a. If yes: What does your child help you do? *Probe to identify what it's like when child helps them (i.e., pleasant, unpleasant, etc.); reasons for letting child help them.*
   b. If no: What are your reasons for not letting your child help?
   c. Role technology plays when cooking with child; role technology COULD play when cooking with child.

5. [A graphic of MyPlate will be emailed to them before the interview.] Please take a look at "attachment 1" on the email I sent you before our interview today. Can you tell me what this is?
   a. What is it for? *Probe to identify what they know about it, if they understand the different parts, etc.*
   b. How many of you use it? *Probe how they use it; reasons for using/not using, benefits and barriers to using.*

6. If you could design a program to help families learn to eat healthy and cook healthy foods:
   Prompts: *Probe to develop specific suggestions/ideas.*
   a. What would you include in it? *Probe topics, skills, etc.*
   b. What would you not include?
   c. Who should attend (e.g., parents, children, ages, etc.)?
   d. What would keep families motivated and engaged?
   e. How would you deliver it (in-person, online, in school, etc.)?
   f. How long would it be?
   g. When should it be offered?
   h. Who should lead the program (e.g., nutritionist/health educator, chef, doctor, promotoro)?
   i. How would you let others know about it?
   j. What should the last meeting include? (e.g., cookoff, celebration, etc.)?

Thank you! What I've heard is [summarize]. Is this correct? Is there anything else you would like to change or add?

Now, let's talk about grocery shopping.

7. Do you grocery shop for your family?

   Prompts:

   a. Where do you grocery shop with your family? *Probe to identify specific information about where – e.g., big box stores, national grocery stores, local grocery stores, corner stores/bodegas/convenience stores, etc.).*
   b. How often do you grocery shop?
   c. Does your child help you grocery shop? *Probe to identify reasons child does/does not help; if yes, probe to identify what child helps with and what it's like for child to help.*
   d. How comfortable are you choosing healthy foods when you grocery shop (1=not comfortable, 2=not sure, 3=comfortable)? *Probe to identify reason or rating they chose and what would increase comfort level if less than 3.*

8. [A graphic of a food label will be emailed to them before the interview.] Please take a look at "attachment 2" on the email I sent you before our interview today. Can you tell me what this is?
   a. What is it for? *Probe to identify what they know about it, if they understand the different parts, etc.*
   b. FOCUS GROUP: How many of you use it? INTERVIEW: Do you use it? *Probe how they use it; reasons for using/not using, benefits and barriers to using it.*

Thank you. *Summarize what you have learned*.

9. Now, we are going to do something a little different. Take a look at the pictures you and your child took for this project. In the next half of our discussion, we are going to talk about your pictures. Take a look at the pictures. For each picture you choose, tell me the number and title you gave it. Now:
   a. Choose one that shows something that makes it hard to eat healthy.
   b. Choose one that shows something that makes it easy to eat healthy.
   c. (if time) Are there other pictures you would like to show us?

   For each picture, ask the following six questions:

   - 1-What do you see?
   - 2-What is happening?
   - 3-How does this relate to your food choices or health
   - 4-Why does this exist (happen)?
   - 5-How can you be empowered by this (how does it make you feel)?
   - 6-What can we do about it?

CLOSING STATEMENT

Thank you so much for speaking with me today! Before we finish, is there anything else you would like to add? [Note: If yes, discuss with them; use probes and prompts as needed to clarify, understand, and expand their responses].

When you and your child both complete an interview with us, I will mail you a $30 [state type of compensation-e.g., money order, cash] as a thank you for your time and your child's time. Is this the address you would like me to send it to [insert address we have for them]?

INTERVIEWER ONLY-POST INTERVIEW

[After the interview is over, and you hang up the phone, please record your impressions of the interview, including anything that may have influenced the quality of the data (e.g., interruptions, crying children, bad connection, disinterest, etc.). Please write the information below and then audio record it at the end of the interview recording; be sure to identify them as "interviewer debriefing" before you start talking.]
